# Revisiting correlation-based functional connectivity and its relationship with structural connectivity

**DOI:** 10.1162/netn_a_00166

**Published:** 2020-12-01

**Authors:** Raphael Liégeois, Augusto Santos, Vincenzo Matta, Dimitri Van De Ville, Ali H. Sayed

**Affiliations:** Institute of Bioengineering, Center for Neuroprosthetics, École Polytechnique Fédérale de Lausanne, Switzerland; Centre for Informatics and Systems, University of Coimbra, Portugal; Department of Radiology and Medical Informatics, University of Geneva, Switzerland; Institute of Bioengineering, Center for Neuroprosthetics, École Polytechnique Fédérale de Lausanne, Switzerland; Centre for Informatics and Systems, University of Coimbra, Portugal; Department of Information and Electrical Engineering and Applied Mathematics, University of Salerno, Italy; Institute of Bioengineering, Center for Neuroprosthetics, École Polytechnique Fédérale de Lausanne, Switzerland; Centre for Informatics and Systems, University of Coimbra, Portugal; Department of Radiology and Medical Informatics, University of Geneva, Switzerland; Institute of Bioengineering, Center for Neuroprosthetics, École Polytechnique Fédérale de Lausanne, Switzerland; Centre for Informatics and Systems, University of Coimbra, Portugal

**Keywords:** Structural connectivity, Functional connectivity, Multimodal modeling, fMRI, Precision matrix, Partial correlation

## Abstract

Patterns of brain structural connectivity (SC) and functional connectivity (FC) are known to be related. In SC-FC comparisons, FC has classically been evaluated from *correlations* between functional time series, and more recently from *partial correlations* or their unnormalized version encoded in the *precision* matrix. The latter FC metrics yield more meaningful comparisons to SC because they capture ‘direct’ statistical dependencies, that is, discarding the effects of mediators, but their use has been limited because of estimation issues. With the rise of high-quality and large neuroimaging datasets, we revisit the relevance of different FC metrics in the context of SC-FC comparisons. Using data from 100 unrelated Human Connectome Project subjects, we first explore the amount of functional data required to reliably estimate various FC metrics. We find that precision-based FC yields a better match to SC than correlation-based FC when using 5 minutes of functional data or more. Finally, using a linear model linking SC and FC, we show that the SC-FC match can be used to further interrogate various aspects of brain structure and function such as the timescales of functional dynamics in different resting-state networks or the intensity of anatomical self-connections.

## INTRODUCTION

The way brain function is shaped by the underlying anatomical substrate is far from understood. Taking advantage of the increasing amount of high-quality anatomical and functional neuroimaging data that has become available in the last decade, various models were proposed to explore this question. The spectrum of models linking brain anatomy and function ranges from simple linear models (Honey et al., 2009; Gu et al., 2015, 2017) to more biologically realistic frameworks involving neural-mass modeling (Deco et al., 2014; Fernández Galán & Galán, 2008; Hansen, Battaglia, Spiegler, Deco, & Jirsa, 2015; Honey, Thivierge, & Sporns, 2010; Messé, Rudrauf, Giron, & Marrelec, 2015; Schirner, McIntosh, Jirsa, Deco, & Ritter, 2018; Wang et al., 2019), stochastic processes (Deco, Jirsa, & McIntosh, 2011; Deco, Senden, & Jirsa, 2012; Deligianni et al., 2011), or advanced dynamical systems tools such as multistability and ghost attractors (Breakspear, 2017; Deco & Jirsa, 2012). The functional connectivity matrix (FC), which encodes the statistical dependencies between brain function in different regions (Friston, 2011), and the structural connectivity matrix (SC), which encodes the strength of anatomical connections between brain regions, were also compared without relying on a model of the interaction between brain structure and function. Beyond observing that the entries of SC and FC matrices are correlated (e.g., Honey et al., 2009; Sporns, Tononi, & Edelman, 2000), it was also found that these matrices share graph-theoretic features (Bullmore & Sporns, 2009; Meunier, Lambiotte, & Bullmore, 2010; Mišić et al., 2016) and that the SC-FC match exhibits temporal fluctuations (Liégeois, Mishra, Zorzi, & Sepulchre, 2015). Recent advances in graph signal processing (Sandryhaila & Moura, 2013; Shuman, Narang, Frossard, Ortega, & Vandergheynst, 2013) have also allowed one to question this relationship from a network theory perspective by linking spectral properties of SC and FC matrices. For example, it was shown that brain function is primarily shaped by anatomical modes computed from the spectral properties of the SC matrix (Abdelnour, Dayan, Devinsky, Thesen, & Raj, 2018; Atasoy, Donnelly, & Pearson, 2016; Huang et al., 2018; Preti & Van De Ville, 2019; Robinson, 2012). Finally, as most studies use functional magnetic resonance imaging (fMRI) data to evaluate FC because of its high spatial resolution, other functional modalities such as electro- or magneto-encephalography were also considered to explore the link between brain anatomy and function (Amico et al., 2017; Finger et al., 2016; Steinmann et al., 2018).

Functional connectivity is classically estimated from the *correlation* between functional time series (Biswal, Yetkin, Haughton, & Hyde, 1995; Buckner et al., 2009; Dosenbach et al., 2007; Power et al., 2011; B. T. Yeo, Krienen, Chee, & Buckner, 2014; Zalesky, Fornito, & Bullmore, 2010). Importantly, the correlation matrix captures both ‘direct’ and ‘indirect’ statistical dependencies. This is in contrast with the *precision* matrix, defined as the inverse of the correlation matrix (Dawid, 1979), that captures only ‘direct’ statistical dependencies by discarding the effects of mediators, as illustrated in Box 1.

Box 1.  Direct and indirect measures of statistical dependence

**Figure 1.**
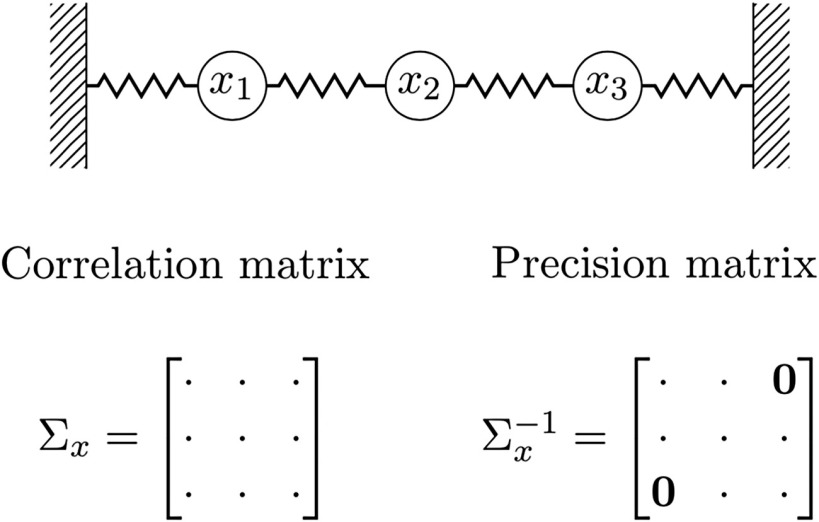
Conceptual difference between the precision and correlation matrices, adapted from MacKay (2006). The mass-spring system represents a set of three coupled masses whose positions along the main axis are denoted by *x*_1_, *x*_2_, and *x*_3_. A non-zero statistical dependence between *x*_1_ and *x*_3_ is encoded in the correlation matrix but not in the precision matrix which encodes *conditional*, or ‘direct’, statistical dependencies.

To illustrate the distinction between ‘direct’ and ‘indirect’ measures of statistical dependence, consider the example of Figure 1 consisting of three masses connected by springs and subject to random excitation.

Denoting *x*_1_, *x*_2_, and *x*_3_ their positions along the main axis, the correlation matrix Σ_*x*_ of *x* = [*x*_1_, *x*_2_, *x*_3_] encodes the classical linear dependence between variables, and every pair of variables exhibits a nonzero correlation. In contrast, the precision matrix, defined as the inverse of the correlation matrix, encodes *conditional* dependencies (Dawid, 1979). For example, ($\Sigma_{x}^{-1}$)_1,3_ encodes the dependence between *x*_1_ and *x*_3_ conditioned on the value of *x*_2_, which corresponds to fixing the mass represented by *x*_2_, and hence its value is zero. In other words, the precision matrix captures ‘direct’ statistical dependencies between variables and discards dependencies arising from intermediate connections captured in the correlation matrix.

The precision matrix or *partial* correlations which can be considered as a normalized version of the precision matrix entries (see Equation 4 in the Methods for details), have been used to estimate FC (Fransson & Marrelec, 2008; Marrelec et al., 2006; Ryali, Chen, Supekar, & Menon, 2012) and were shown to provide better prediction scores than correlation-based FC in some cases (Dadi et al., 2019; Pervaiz, Vidaurre, Woolrich, & Smith, 2020; S. M. Smith et al., 2013). In the context of SC-FC comparisons, FC has also classically been evaluated from the correlation between functional time series (e.g., Honey et al., 2009). However, since SC matrices encode ‘direct’ anatomical connections, the precision matrix, or partial correlations, appears as a more natural metric than the correlation matrix to estimate FC when comparing the properties of SC and FC matrices. For example, M. van den Heuvel, Mandl, Luigjes, and Hulshoff Pol (2008) and Lefort-Besnard et al. (2018) estimated FC from partial correlations (resp., the precision matrix) to explore the SC-FC link within the default mode network, and Huang and Ding (2016) interrogated this link in a network composed of four nodes using different FC estimates such as correlation, partial correlations, and Granger causality (Wu, Liao, Stramaglia, Chen, & Marinazzo, 2013). While the theoretical advantage of using ‘direct’ FC measures of statistical dependencies when exploring the SC-FC link has been documented (Deligianni et al., 2011), their use remains limited, a potential reason for this being that their estimation involves a matrix inversion that yields noisy estimates when a limited amount of functional data is available (S. M. Smith, Vidaurre, et al., 2013).

Considering the recent improvements in structural and functional neuroimaging data quality, we propose to revisit the use of different FC metrics in the context of SC-FC comparisons. We first recall the theoretical arguments supporting the use of ‘direct’ measures of statistical dependencies to evaluate FC. We then compute the match between SC and four different estimates of FC computed from varying lengths of fMRI data: correlation-based FC, which captures direct and indirect statistical dependencies; precision-based FC, which captures only direct statistical dependencies; regularized precision-based FC, which allows more stable precision matrix estimation; and autoregressive-based FC, which captures dynamic statistical dependencies (Liégeois, Laumann, Snyder, Zhou, & Yeo, 2017). We finally explore the SC-FC link in different resting-state networks and discuss how a simple brain structure-function model can be adapted to better interpret the nature of this link in terms of functional dynamics as well as the underlying brain anatomy. Overall, beyond recalling the theoretical advantage of ‘direct’ FC measures, our work provides empirical evidence of this advantage and shows how the nature of the SC-FC link can be used to better characterize both brain anatomy and function.

## METHODS

### Data

We used data from 100 unrelated subjects from the Human Connectome Project (HCP) 1200-release comprising resting-state functional magnetic resonance imaging (fMRI) and diffusion-weighted scans of young (ages 22–35) and healthy participants (Van Essen et al., 2013). Data were acquired on a 3-T Siemens Skyra scanner using a multiband sequence. Functional images have a repetition time (TR) of 0.72 sec and a 2-mm isotropic spatial resolution. For each subject, four 14.4 min runs (1,200 frames) of functional time series were acquired (S. M. Smith, Beckmann, et al., 2013). Functional volumes underwent a spatial smoothing by a 5-mm isotropic Gaussian kernel using SPM8 and the first 10 volumes were discarded, resulting in 1,190 time points for each run. The fMRI time series were detrended, and we regressed out six motion parameters, average cerebrospinal fluid signal, and white matter signal. From these voxel-level time series, we computed the average signal in *N* = 360 regions of interest (ROIs) using the multimodal parcellation of Glasser et al. (2016). Starting from these ‘original’ time series, we also generated ‘filtered’ time series by performing a 0.01–0.15 *Hz* band-pass filtering. Finally, ‘deconvolved’ time series were generated following Gaudes, Karahanoğlu, Lazeyras, and Ville (2012) and *λ*_1_ = 4 in order to explore the impact of the hemodynamic response function on our results. For each run, all time series were individually centered and normalized to unit variance in order to allow concatenation of time series from different runs or subjects. We used MRtrix3 (http://www.mrtrix.org) to analyze diffusion-weighted scans and applied multishell multitissue response function estimation with constrained spherical deconvolution. A tractogram with 10^7^ streamlines was generated using the second-order integration over Fiber Orientation Distributions (iFOD2) probabilistic algorithm and was then filtered using SIFT such that the streamline densities match the FOD lobe integrals (R. E. Smith, Tournier, Calamante, & Connelly, 2013). Finally, we computed the number of fibers connecting every pair of the 360 ROIs defined in Glasser et al. (2016), normalized by the volumes of the connected ROIs (i.e., departing and ending ROIs), to generate individual structural connectivity matrices. The group structural connectivity matrix (Supporting Information Figure S7) was obtained by averaging the subjects’ structural connectivity matrices.

### Four FC Measures

We computed four multivariate measures of statistical dependencies from the fMRI time series parcellated into 360 ROIs. The first FC metric is the classical Pearson correlation matrix between fMRI time series that encodes direct and indirect statistical dependencies (Figure 1). The second FC metric consisted of the precision matrix, that is, the inverse of the correlation matrix, which captures conditional, or ‘direct’ statistical dependencies (Dawid, 1979). Since inverting the correlation matrix might be an unstable operation, especially when it is computed from few time points (Brier, Mitra, McCarthy, Ances, & Snyder, 2015), we also considered a regularized version of the precision matrix. Specifically, we used the Tikhonov regularization that consists in adding a full-rank regularization term to the correlation matrix before performing the inversion. The regularization term is *γ* ⋅ 𝕀, where 𝕀 is the identity matrix and *γ* is a parameter that was optimized by minimizing the distance between regularized subject precision matrices and the unregularized group precision matrix as in Pervaiz et al. (2020) (see details in Supporting Information Methods). Finally, in order to explore whether including dynamical properties of fMRI time series in FC could improve the match with SC, we considered a fourth FC metric relying on a first-order multivariate autoregressive model of the fMRI time series, as this model was found to concisely capture fMRI dynamics (Liégeois et al., 2017). Autoregressive-based FC, or AR-based FC, is defined as the symmetric part of this autoregressive model parameter in order to make the comparison with SC, which is by definition symmetric, more meaningful. When data from different runs or subjects are concatenated, the autoregressive model is identified from the concatenated time series, neglecting points corresponding to transitions between different runs or subjects (Casorso et al., 2019). Group-level matrices corresponding to these FC metrics are shown in Supporting Information Figure S7.

### Computing the Match Between SC and FC

Following methodology in previous work exploring the link between brain function and anatomy (e.g., Honey et al., 2009), the match between SC and FC matrices, denoted by *ρ*, was evaluated from the correlation between their vectorized upper triangular parts:

$$\rho =corr(FC^{⊳},SC^{⊳}),$$ (1)

where the operator ⊳ transforms a matrix into a vector containing its upper triangular entries. The diagonal entries of FC and SC matrices are not used by this operator as the diagonal entries of some FC measures do not encode relevant information (e.g., they are all equal to 1 for correlation-based FC). The SC-FC match *ρ* was computed using all off-diagonal SC and FC entries. Supporting Information Figure S3 shows that our main findings still hold when the SC-FC match is computed using only nonzero SC (and corresponding FC) entries (Supporting Information Figure S3A–C), or when SC entries are not normalized by the corresponding ROIs’ volumes (Supporting Information Figure S3D). Finally, we used absolute values of FC matrices to compute the match with SC in Equation 1 because, in the same way SC cannot be negative, the sign of FC entries is not meaningful in terms of ‘strength’ of a statistical dependence.

**Figure 2.**
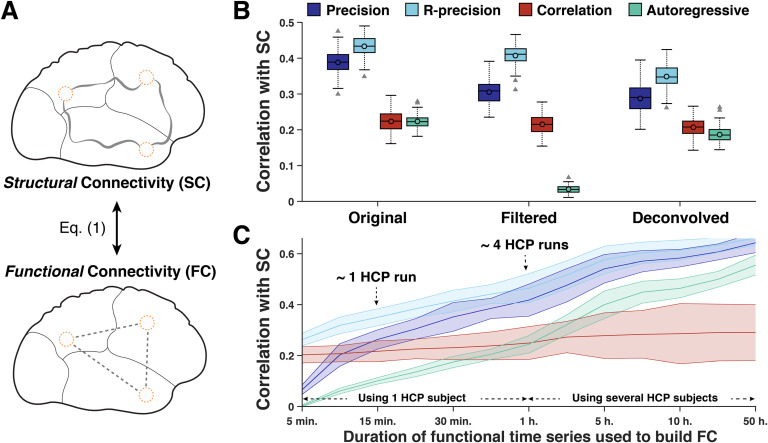
Structural connectivity is best reflected by functional connectivity (FC) evaluated from the (regularized) precision matrix, that is, precision-based FC. (A) Structural connectivity (SC) encodes the strengths of the anatomical links (i.e., white matter tracks) between pairs of cortical regions. Functional connectivity (FC) encodes the statistical dependence between functional activity in pairs of cortical regions. (B) Correlation between SC and FC evaluated using Equation 1 and estimating FC from the precision matrix (dark blue), the regularized precision matrix (‘R-precision’, light blue), the correlation matrix (red), and the autoregressive matrix (green) in original, filtered, and deconvolved fMRI time series using the four runs for each subject (∼1 hour of scanning). Bar plots represent distributions over the 100 subjects. (C) Mean and standard deviation of correlation between SC and FC as a function of the duration of the original time series used to build FC.

In order to evaluate the SC-FC match when using more data than what is available for a single subject (i.e., ∼1 hour), we concatenated data of different subjects (e.g., S. M. Smith, Vidaurre, et al., 2013). More precisely, in order to evaluate the SC-FC match when using several hours of data, we concatenated functional time series coming from *K* randomly chosen subjects where *K* is chosen so as to match the required number of hours. FC metrics are computed from these concatenated time series and the SC matrix is the average of the SC matrices of these *K* subjects. This procedure is repeated 100 times in order to estimate the mean and standard deviation of the match between SC and FC using several hours of functional time series (e.g., Figure 2C). Note that when considering, for example, 20 hours of time series, the corresponding 100 samples are not independent as the total amount of data available to generate 100 samples of 20 hours is approximately 92 hours. In that case, the sample standard deviation of the SC-FC match underestimates the true standard deviation and we used the Jackknife framework to correct for this in Figure 2C (Efron & Tibshirani, 1986).

### Beyond Intuition: A Model of Brain Structure-Function Interactions

Intuition that the SC matrix should be compared to precision-based FC rather than correlation-based FC can be theoretically supported by relying on an Ornstein–Uhlenbeck based model (Timme & Casadiego, 2014; Uhlenbeck & Ornstein, 1930) to represent brain structure-function interactions (Fernández Galán & Galán, 2008; Gilson, Moreno-Bote, Ponce-Alvarez, Ritter, & Deco, 2016; Gu et al., 2017):

$${\dot{y}}_{t}=B\cdot y_{t}+x_{t},$$ (2)

where **y**_*t*_ is a vector encoding the functional signal measured in all ROIs, **x**_*t*_ is a vector encoding the driving input noise in all ROIs, and *B* is a negative semidefinite matrix encoding the connections between all pairs of ROIs. It can be shown that when the driving noise **x**_*t*_ can be represented by white Gaussian noise, we have Σ ∝ *B*^−1^, or equivalently Σ^−1^ ∝ *B*, where Σ is the correlation matrix of the signal **y**_*t*_ (e.g., Oku & Aihara, 2018). In other words, in a system where the link between the dynamics encoded in **y**_*t*_ and the underlying structure *B* is governed by Equation 2, the entries of the structural matrix *B* and the precision matrix of **y**_*t*_ are perfectly correlated.

The model of Equation 2 assumes linearity of brain structure-function interactions and is therefore not expected to fully reflect the complex nature of functional dynamics (e.g., Deco, Jirsa, Robinson, Breakspear, & Friston, 2008; Stephan et al., 2008). Yet, it can be used to further characterize the nature of functional dynamics and the underlying anatomy. We show in the Supporting Information Methods that when the timescale of driving dynamics **x**_*t*_ is significantly slower than the information exchange through the underlying anatomical backbone (in that case **x**_*t*_ cannot be modeled by white noise), we have Σ ∝ *B*^−2^, and the optimal estimator of the underlying graph structure *B* is Σ^−$\frac{1}{2}$^ (Supporting Information Equation S6). More precisely, the exponent value *β* giving the best match between Σ^*β*^ and *B* encodes information about the relative timescales of internal driving noise and information exchange on the structural backbone in Equation 2. In Figure 4 we show the optimal value of *β* at the whole-brain level and in different resting-state networks. To this end we computed the match between the corresponding SC and FC matrices, where FC is evaluated from Σ^*β*^, Σ is the functional correlation matrix, and *β* is varied between − 3 and 3. The case *β* = 1 corresponds to using correlation-based FC, and *β* = −1 corresponds to using precision-based FC. Finally, note that in the case where the structure-function link can be represented by an Ising model, the optimal estimator of the underlying graph structure is obtained by thresholding the entries of the empirical correlation matrix Σ (Montanari & Pereira, 2009, Theorem 1.1).

Model 2 can also be used to further characterize brain anatomy. Indeed, the structural matrix *B* can be expressed as:

$$B\overset{\Delta }{=}D+S,$$ (3)

where *D* is a diagonal matrix and *S* contains only zeros on its diagonal. Following Equation 2, it can be seen that the entries of *D* are proportional to the diagonal entries of the precision matrix Σ^−1^, which allows one to estimate the relative intensity of anatomical self-connections within each ROI (Figure 5).

Finally, note that the precision matrix and partial correlations are linked as follows:

$$\sigma_{i,j}=-\frac{{\left(\Sigma^{-1}\right)}_{i,j}}{{\left(\Sigma^{-1}\right)}_{i,i}{\left(\Sigma^{-1}\right)}_{j,j}},$$ (4)

where *σ*_*i*,*j*_ is the partial correlation between variables *i* and *j*. Hence, partial correlations also encode ‘direct’ connections between the variables and can be seen as a standardized, or normalized, version of the information contained in the precision matrix (Whittaker, 2009). We preferred using the precision matrix as a measure of ‘direct’ statistical dependencies mainly because the diagonal entries of this matrix encode information on the anatomical self-connections within each variable, as explained here above (see Discussion for more details).

## RESULTS

### Precision-Based FC Best Captures SC

We first explore the correlation between SC and FC evaluated from four metrics of fMRI time series: (i) correlation, (ii) precision, (iii) regularized precision, and (iv) autoregressive matrices.

It can be seen from Figure 2B that (regularized) precision-based FC has the best match with the underlying anatomy encoded by the SC matrix. Using the original (i.e., unfiltered) fMRI time series, the correlation between SC and precision-based FC is 0.39 on average over the 100 subjects and 0.44 in the regularized case, whereas it is 0.24 and 0.23 when using correlation-based FC and AR-based FC, respectively. A similar trend is observed when using filtered fMRI time series or when the four FC metrics are computed from deconvolved time series. Note that the SC-FC match using correlation-based FC (0.24) is smaller than what was observed in other studies using the same FC metric (e.g., Honey et al., 2009) found SC-FC coupling values between 0.3 and 0.5). This difference might result from the way the SC-FC match is computed: for example, considering only non-zero SC entries to evaluate the SC-FC match, as in Honey et al. (2009), yields coupling values around 0.37 (Supporting Information Figure S3B and S3C). Then, the poor performance of AR-based FC in the filtered case is expected because autoregressive models perform poorly on filtered time-series, as observed in previous work (e.g., Casorso et al., 2019). Finally, the results in the ‘deconvolved’ case suggest that removing the effect of the hemodynamic response function does not affect the advantage of precision-based FC over the two other FC metrics.

Figure 2C shows the SC-FC correlation as a function of the duration of the fMRI time series used to compute the four FC metrics. When few samples are available, AR-based and precision-based FC measures perform poorly because they both involve the inversion of a matrix that is close to rank deficiency (Stoica & Moses, 2005). In that case, the best match to SC is provided by the regularized precision matrix, which performs better than correlation-based FC even when using 5 minutes of functional data (with a TR of 0.72 sec). When increasing the amount of data to estimate FC metrics, the impact of regularizing the precision matrix estimate decreases, and when 1 hour of data is used both regularized and unregularized precision-based FC outperform correlation-based FC (*p* < 10^−6^, two-tailed *t* test). This case corresponds to using the four runs for each subject and is detailed in Figure 2B. More important is the fact that when concatenating data from different subjects for an increasing number of hours, the match between (regularized) precision-based FC and SC keeps increasing to reach values of *ρ* > 0.60, which confirms the fact that the precision matrix is a more meaningful measure of FC for SC-FC comparisons. When using AR-based FC, the match to SC follows a similar trend even if *ρ* is systemically smaller than for precision-based FC. On the contrary, the average match between correlation-based FC and SC tends to plateau around *ρ* = 0.28 when a few hours of functional data are used, while *ρ* = 0.20 when using 5 minutes of data. We finally note that using the same number of fMRI time points but with a larger repetition time (TR), precision-based FC and correlation-based FC estimates have a better match to SC, whereas AR-based FC is less correlated with SC (Supporting Information Figure S5). This is explained by the fact that fMRI time points carry less redundant information when the sampling period TR increases, which is beneficial for the estimation of the correlation and precision matrices, but penalizes the estimation of the AR-based metric because it exploits autocorrelation of time series. Obviously, considering longer repetition times also comes at the price of longer scanning sessions for a given number of time points.

### SC-FC Match is Stronger in Primary Sensory and Motor Networks

**Figure 3.**
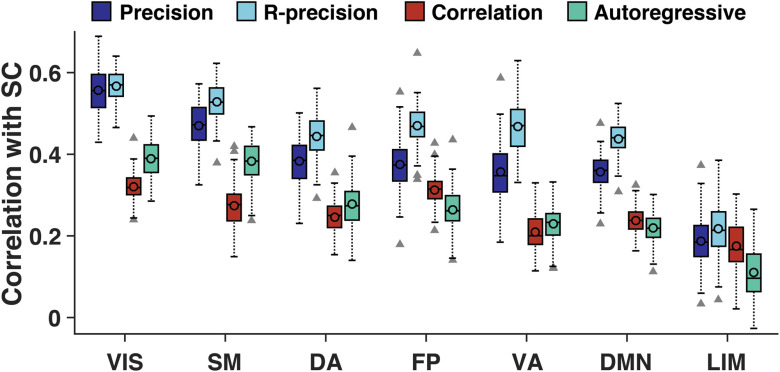
Correlation between SC and precision-based FC (dark blue), regularized precision-based FC (‘R-precision’, light blue), correlation-based FC (red) and the AR-based FC (green) computed from unfiltered fMRI time series in seven resting-state networks of B. T. T. Yeo et al. (2011): visual (VIS), somato-motor (SM), dorsal-attentional (DA), fronto-parietal (FP), limbic (LIM), and default mode networks (DMN). Bar plots represent distributions over the 100 subjects; mean (median) is represented by the circle (horizontal) line, rectangles cover the first to third quartiles, dotted lines cover 1.5 times the rectangle range, and elements out of this range are represented by gray triangles.

In Figure 3, we explore the nature of the SC-FC correlation in seven resting-state networks defined in B. T. T. Yeo et al. (2011) using four runs for each subject. In the visual network the group-averaged correlation between SC and precision-based FC is 0.57, whereas it is 0.19 in the limbic network. Networks are shown from left to right with decreasing values of the match between SC and precision-based FC. It can be seen that primary sensory and motor networks exhibit a better SC-FC match than networks that are involved in more abstract cognitive functions such as the default mode network.

### On the Nature of SC-FC Dynamics

**Figure 4.**
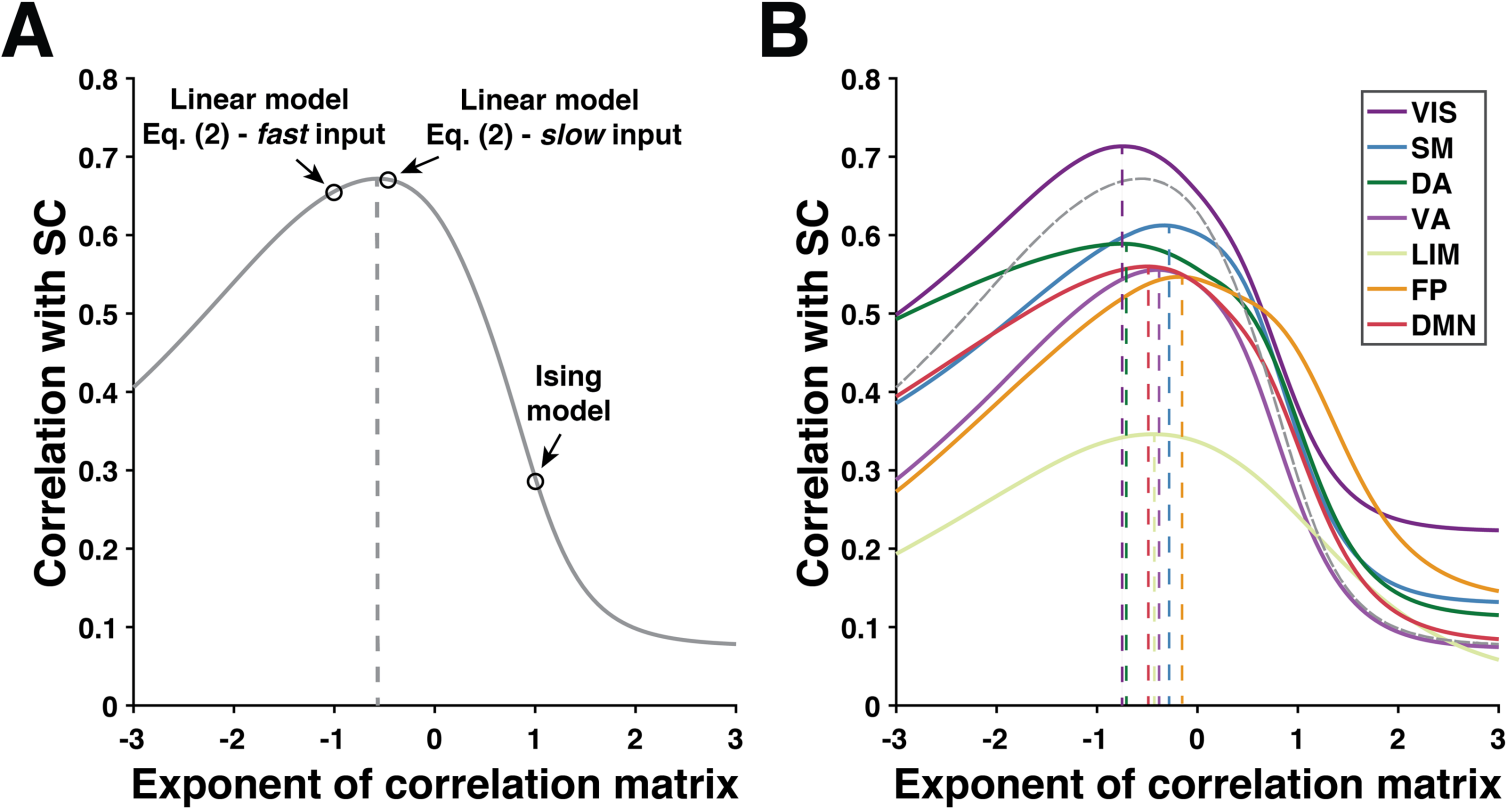
Correlation between SC and FC evaluated from various exponents of the correlation matrix of fMRI time series concatenated over the 100 HCP subjects. (A) When using whole-brain data, the optimal exponent is around −0.7. (B) Optimal exponent when using only data in the seven resting-state networks of B. T. T. Yeo et al. (2011).

In previous results we have considered the match between SC and correlation-based FC, which uses the correlation matrix of fMRI time series as an FC marker, and precision-based FC, which relies on the inverse of the fMRI correlation matrix. In other words, we compute the match between SC and FC using two different exponents of the fMRI correlation matrix (1 and −1, respectively) to estimate FC. In Figure 4, we explore the SC-FC match when FC is evaluated using various exponents of the fMRI correlation matrix.

It can be observed from Figure 4A that at the whole-brain level, the exponent value of the fMRI matrix that provides the best match to SC is around −0.7. This optimal value is also found to be different for different resting-state networks, which also finds an interpretation in terms of timescales of the driving functional dynamics happening in different networks. Indeed, following Supporting Information Equations S5–S7 and Figure S1, results of Figure 4B suggest that internal driving dynamics in the visual network happen at faster timescales than internal dynamics in other networks because the optimal exponent in the visual network is the closest to −1. Note that these properties seem to be reproduced at the subject level when using a typical acquisition time around 15 minutes (Supporting Information Figure S6). Finally, we explored the link between the whole-brain optimal exponent of each subject and behavioral measures including bodily, cognitive, and task performance measures but did not find significant links (Supporting Information Figure S4).

### Intensity of Anatomical Self-Connections

**Figure 5.**
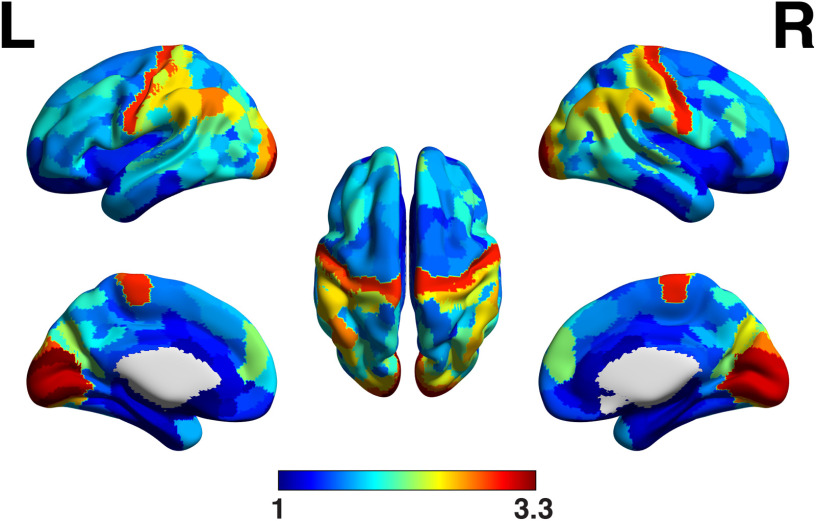
Diagonal entries of the precision matrix of fMRI timeseries suggest that stronger anatomical self-connections (i.e., ‘self-loops’) take place within the visual and sensory-motor regions.

When we compare SC and FC matrices, we only use the upper triangular parts of the corresponding matrices, neglecting their diagonal entries. We note from Equation 3 that the diagonal entries of the structural matrix are accessible from the diagonal entries of the FC estimator. These values are shown in Figure 5 when the precision matrix is used to estimate FC (similar results are obtained when the optimal FC estimator is used, i.e., Σ^−0.7^). It can be seen that visual and motor areas exhibit stronger anatomical self-connections.

## DISCUSSION

The match between FC, usually evaluated from the correlation matrix of fMRI time series, and SC has been highlighted in various studies (e.g., Honey et al., 2009; Messé et al., 2015; M. P. van den Heuvel, Mandl, Kahn, & Hulshoff Pol, 2009). We show that estimating FC from the precision matrix allows more meaningful SC-FC comparisons, while also providing further insights into the nature of functional dynamics.

### Conditions to Estimate the Precision Matrix

We have seen that the precision matrix provides a more natural FC estimate for SC-FC comparisons than correlation. Precision-based FC was also shown to provide better prediction scores than correlation-based FC for some diseases and phenotypic measures (Dadi et al., 2019; Pervaiz et al., 2020; S. M. Smith, Vidaurre, et al., 2013). However, one important drawback of the precision matrix as compared to the correlation matrix is its estimation. Indeed, the precision matrix relies on the inversion of the correlation matrix which might be an unstable operation when not enough time points, as compared to the number of variables, are available to estimate the correlation matrix (Brier et al., 2015; S. M. Smith, Vidaurre, et al., 2013). This results in poor precision matrix estimates when using few data points (e.g., dark blue curve in Figure 2C). To overcome this limitation we used Tikhonov regularization, which allows for better precision matrix estimates, especially when using few data points (light vs. dark blue curve in Figure 2C). Our results suggest that the advantage of using (regularized) precision-based FC over correlation-based FC appears when using at least as many time points as variables, that is, ROIs or voxels. This precludes using the precision matrix in voxel-based studies, while in atlas-based studies a few hundred time points should be sufficient to estimate precision-based FC matrices having a better match to SC than correlation-based FC. Note that other regularization approaches have been proposed such as *L*_1_ regularization (Friedman, Hastie, & Tibshirani, 2008; S. M. Smith et al., 2011; Varoquaux, Gramfort, Baptiste Poline, & Thirion, 2010), Ledoit–Wolf shrinkage (Deligianni, Centeno, Carmichael, & Clayden, 2014), or population-based shrinkage (Rahim, Thirion, & Varoquaux, 2019). Future work will explore whether such approaches provide better precision matrix estimates, and would ideally also need to account for the type of noise inherent to fMRI signals (Liu, 2016; Niazy, Xie, Miller, Beckmann, & Smith, 2011). Then, similar limitations apply to the estimation of autoregressive models from fMRI time series as this identification also relies on a matrix inversion (Stoica & Moses, 2005). The fact that the match between AR-based FC and SC is systematically lower than when using precision-based FC (Figure 2B and 2C) suggests that while AR-based FC captures behaviorally relevant functional dynamics (Liégeois et al., 2019), these dynamics might be too complex to be reflected by the underlying brain anatomy. Finally, quite intriguing is the fact that the match between SC and (regularized) precision-based FC keeps increasing even when using up to 50 hours of functional time series by concatenating data from several subjects (Figure 2C). Besides further supporting precision-based FC as a more meaningful metric to be compared to SC, this observation raises other questions. First, it is unclear whether this trend results from averaging effects that remove individual specificities or only from improved precision matrix estimates. One way to test this is to compute the match between precision-based FC and SC using 10, 20, or 50 hours of functional data acquired on a single subject and compare these results to the ones presented in Figure 2C. Such amounts of functional data are not available in the HCP dataset for a single subject, hence we performed this experiment using 1 hour of functional data. Our results suggest that averaging structural and functional connectomes indeed slightly increases the SC-FC match (Supporting Information Figure S8). Second, the nature of the SC-FC coupling when using more than 50 hours of data could be further explored. When concatenating data from all 100 subjects (∼92 hours, results not shown in Figure 2C) the match between precision-based FC and SC is 0.655, as compared to 0.643 when using 50 hours of data and 0.619 when using 25 hours of data (Figure 2C, dark blue curve). In other words, while the match between precision-based FC and SC keeps increasing when using more than 50 hours of functional data, the rate of increase gets lower, which might indicate the presence of an upper bound to this match.

### Related Metrics and Methods

The precision matrix and partial correlations are linked through Equation 4. We preferred using the precision matrix for two reasons. Most importantly, unlike the partial correlation matrix, the diagonal entries of the precision matrix are in general different from one, which might carry information on the nature of the internal properties of each variable, as illustrated in Figure 5. Then, the signs of the entries of the precision and partial correlation matrices are opposite, but we believe that this sign is not meaningful in terms of the strength of a statistical dependence, which is why we use the absolute values of FC entries when comparing SC and FC matrices. We note that when not taking the absolute values of the entries in the FC matrices, the SC-FC match is strongest when FC is evaluated from −Σ^−1^, as suggested from Supporting Information Equation S5. Our results also find a deeper echo in recent work using graph signal processing tools to show that correlation-based FC can be expressed as a sum of anatomical ‘modes’ derived from SC eigenvectors (Abdelnour et al., 2018; Atasoy et al., 2016). Indeed, since the inversion operation amounts to invert the eigenvalues of a matrix while preserving its eigenvectors, finding a strong match between precision-based FC and SC matrices suggests that both precision-based and correlation-based FC can be approximated by a weighted sum of eigenvectors derived from SC. Similarly, finding an optimal SC-FC match using an exponent of the correlation matrix around −0.7 is coherent with the inverse squared relationship found between eigenvalues of SC and correlation-based FC in Robinson, Sarkar, Pandejee, and Henderson (2014) or the negative exponential relationship identified in Abdelnour et al. (2018).

Graph theory metrics have also been widely used to characterize organization of FC matrices as well as their links to SC (Bullmore & Sporns, 2009; Meunier et al., 2010; Mišić et al., 2016). This is done by building a graph whose edges are defined by the entries of FC matrices that are again most often estimated from the correlation between time series (Hallquist & Hillary, 2019). However, most graph metrics were defined and are meaningful only if applied on graphs with edges encoding conditional or ‘direct’ statistical connections (e.g., Koller & Friedman, 2009; Whittaker, 2009). As an illustration, consider computing a path-length metric on graphs defined from the mass-spring example of Figure 1. We expect this metric to be largest between variables representing the first and the third mass. This is the case when building the graph from the corresponding precision matrix, but might not be the case when using the correlation matrix. Therefore, beyond being more relevant to explore the SC-FC link, precision-based FC might also be considered in other cases such as the definition of functional graphs.

### What SC-FC Interactions Tell Us About Brain Function and Structure

Results of Figure 3 show that the SC-FC match is higher in primary sensory and visual networks and lower in networks involved in more complex cognitive functions such as the default mode network. In other words, the simple linear model linking brain anatomy and function presented in Equation 2 better explains brain structure-function interactions happening in primary sensory networks than in the default mode network where functional dynamics are likely to be more complex. Beyond being characterized by a better SC-FC match, sensory networks were also found to exhibit faster functional dynamics (Figure 4) which might reflect the nature of sensory inputs processing. These results are consistent with recent findings showing that the degree of decoupling between structure and function in a brain region reflects the complexity of the cognitive functions in which the region is involved (Preti & Van De Ville, 2019). Then, when ordering resting-state networks following a decreasing value of SC-FC match as in Figure 3, the sequence of networks almost perfectly matches the main gradient of functional cortical organization identified by Margulies et al. (2016), suggesting that the underlying brain anatomy plays a key role in shaping this functional gradient. Finally, in Figure 5 we show that precision-based FC can also be used to infer the intensity of anatomical self-connections within each ROI. The distribution of these connections shown in Figure 5 strongly resembles the one found by Wang et al. (2019) who identified them, among other parameters, by inverting a nonlinear large-scale circuit model, thereby further supporting the relevance of precision-based FC to explore SC-FC interactions.

### Limitations and Future Directions

Using the precision matrix to evaluate FC allows more meaningful comparisons with the underlying anatomy. This measure relies on the inversion of the correlation matrix and therefore only captures linear statistical dependencies. Evaluating FC using nonlinear measures of statistical dependencies (Chai, Walther, Beck, & Fei-Fei, 2009; David, Cosmelli, & Friston, 2004; Marinazzo, Liao, Chen, & Stramaglia, 2011) could help further bridge the gap between SC and FC matrices. Alternative measures of functional dependencies could also exploit the spectral structure—or equivalently the autocorrelation structure because of the Wiener–Khintchine theorem (Wiener, 1930)—of the time series. While the autoregressive metric used in this work only captures first-order autocorrelation information on the time series, one might exploit the whole time series correlation structure from their power spectral density whose inverse also captures conditional statistical dependencies (Brillinger, 2001). In this case the estimation issue would be magnified by the fact that the entries of the matrix to be inverted are functions of the frequency instead of scalars (Liégeois et al., 2016), thereby also making the comparison to SC less straightforward. Then, we proposed an FC metric that only captures ‘direct’ statistical dependencies in order to make the comparison to SC more relevant. Another way to explore the SC-FC coupling would be to derive anatomical communication measures from the structural connectome (e.g., using shortest path or percolation measures) that encode both direct and indirect connections (Avena-Koenigsberger, Misic, & Sporns, 2017; Goñi et al., 2014), thereby making the comparison with correlation-based FC more relevant. Finally, we have focused in this work on FC metrics derived from fMRI time series. While the theoretical arguments presented here are general and do not rely on fMRI specificities, further work is required to explore the extent to which our results also apply when FC is evaluated from alternative functional neuroimaging modalities such as electro- or magnetoencephalography.

## CONCLUSION

This work revisits the use of ‘direct’ statistical dependencies metrics such as partial correlations or the precision matrix to evaluate FC when exploring the SC-FC match and provides practical guidelines on the amount of data required to reliably estimate these metrics. For classical atlas-based approaches, our results suggest that the advantage of (regularized) precision-based FC over correlation-based FC is significant when the functional timeseries used to compute these metrics contain more time points than variables (i.e., ROIs), which typically corresponds to a 5-minutes acquisition. Moreover, the SC-FC match can be used to further characterize functional dynamics and the underlying anatomical backbone. Overall, our work presents a theoretical and practical motivation for using (regularized) precision-based FC in the context of SC-FC comparisons, while also providing tools to interpret the nature of this link.

## ACKNOWLEDGMENTS

The authors thank Alessandra Griffa, Maria Giulia Preti, and Enrico Amico for the helpful discussions and suggestions. Data and ethics were provided by the Human Connectome Project, WU-Minn Consortium (Principal Investigators: David Van Essen and Kamil Ugurbil; 1U54MH091657) funded by the 16 NIH Institutes and Centers that support the NIH Blueprint for Neuroscience Research; and by the McDonnell Center for Systems Neuroscience at Washington University.

## SUPPORTING INFORMATION

Supporting information for this article is available at https://doi.org/10.1162/netn_a_00166.

## AUTHOR CONTRIBUTIONS

Raphael Liegeois: Conceptualization; Investigation; Data curation; Formal analysis; Methodology; Supervision; Writing - Original Draft; Writing - Review & Editing. Augusto Santos: Conceptualization; Investigation; Formal analysis; Methodology; Writing - Review & Editing. Vincenzo Matta: Conceptualization; Investigation; Formal analysis; Methodology; Writing - Review & Editing. Dimitri Van De Ville: Conceptualization; Methodology; Supervision; Writing - Review & Editing. Ali H. Sayed: Conceptualization; Methodology; Supervision; Writing - Review & Editing.

## FUNDING INFORMATION

Torsten Moeller, Austrian Science Fund, award ID: 20CH21 174081. Dimitri Van De Ville, Swiss National Science Foundation, award ID: 20CH21 174081. Jean-Daniel Fekete, Agence Nationale de la Recherche, award ID: 20CH21 174081.

## TECHNICAL TERMS

Functional connectivity (FC): Statistical dependencies between brain function in different brain regions.
Structural connectivity (SC): Strength of anatomical connections between brain regions.
Correlation matrix: Matrix encoding Pearson correlation values between pairs of variables in a multivariate setting.
Precision matrix: Inverse of the correlation matrix; its entries only encode ‘direct’ statistical dependencies.
Partial correlation: Normalized version of the precision matrix entries thereby only encoding ‘direct’ statistical dependencies.
Indirect statistical dependencies: Statistical dependencies that arise from the presence of shared connections.
Direct statistical dependencies: Statistical dependencies that discard the effect of mediators.
Pearson correlation: Common correlation measure that encodes both ‘direct’ and ‘indirect’ statistical dependencies.
