# Supplementary material for: Revisiting correlation-based functional connectivity and its relationship with structural connectivity
Source: Netw Neurosci. 2020 Dec 1;4(4):1235–51. doi: 10.1162/netn_a_00166 (PMC7781609; doi:10.1162/netn_a_00166)
Supplement: Supplementary file 1 [file netn-04-1235-s001.pdf]

Liegeois, R., Santos, A., Matta, V., Van De Ville, D., & Sayed, A. H. (2020). Supporting information for "Revisiting correlation-based functional connectivity and its relationship with structural connectivity." *Network Neuroscience, 4*(4), 1235–1251.
https://doi.org/10.1162/netn_a_00166

SUPPLEMENTARY MATERIAL - METHODS

Linking observed correlation and latent driving noise

The solution of the linear model of brain structure-function interactions defined in Eq. (2) is:

$$\mathbf{y}_t = e^{Bt} \int_0^t e^{-B\tau} \mathbf{x}_\tau d\tau, \tag{S1}$$

where B encodes anatomical connections (Eq. (3)), $\mathbf{y}_t$ denotes observed brain function at time t, $\mathbf{x}_t$ is the driving noise at time t, and we have $\mathbf{y}_0 = 0$. Let us now assume that $\mathbf{x}_t$ evolves in a stepwise fashion, with a certain unknown step h, i.e., $\mathbf{x}_t = \boldsymbol{\xi}_k$ for all $t \in [(k-1)h, kh]$ where the sequence of random values $\{\boldsymbol{\xi}_k\}$ fulfills the (stepwise) whiteness condition $\mathbb{E}[\boldsymbol{\xi}_k \boldsymbol{\xi}_{k'}^\top] = \sigma_x^2 \delta_{kk'}$. Let us now focus on a time epoch $t = nh + \Delta$, where n is an integer and $0 < \Delta < h$. Under the aforementioned model, we can apply (S1) to obtain:

$$\begin{aligned} \mathbf{y}_t &= e^{Bt} \left[\sum_{k=1}^n \boldsymbol{\xi}_k \int_{(k-1)h}^{kh} e^{-B\tau} d\tau + \boldsymbol{\xi}_{n+1} \int_{nh}^t e^{-B\tau} d\tau \right] \\ &= -e^{B\Delta} B^{-1} \sum_{k=1}^n \left(e^{B(n-k)h} - e^{B(n-k+1)h} \right) \boldsymbol{\xi}_k - \left(I - e^{B\Delta} \right) B^{-1} \boldsymbol{\xi}_{n+1} \\ &= -e^{B\Delta} B^{-1} \sum_{m=0}^{n-1} e^{Bmh} (I - e^{Bh}) \boldsymbol{\xi}_{n-m} - \left(I - e^{B\Delta} \right) B^{-1} \boldsymbol{\xi}_{n+1}, \end{aligned} \tag{S2}$$

where $m = n - k$ and we have used the fact that B^{-1} and e^{Bt} commute. Using the whiteness condition, we can now compute the covariance of $\mathbf{y}_t$, denoted by $\Sigma(\Delta)$, as:

$$\Sigma(\Delta) = \sigma_x^2 B^{-2} \left[e^{2B\Delta} (I - e^{Bh})^2 (I - e^{2Bh})^{-1} + \left(I - e^{B\Delta} \right)^2 \right], \tag{S3}$$

where we have repeatedly applied commutativity of the pertinent matrices, the cross-terms are annihilated due to the whiteness condition, and we have considered n sufficiently large in order for the sum of the following geometric matrix progression to be expressed as: $\sum_{m=0}^{n-1} e^{2Bmh} \approx \sum_{m=0}^{\infty} e^{2Bmh} = (I - e^{2Bh})^{-1}$, where we have also used the fact that matrix B is negative semidefinite. The average covariance of the observed signal can then be obtained by computing:

$$\begin{aligned} \Sigma &= \frac{1}{h} \int_0^h \Sigma(\Delta) d\Delta \\ &= \sigma_x^2 B^{-2} \left[-\frac{B^{-1}}{2h} \left(I - e^{Bh} \right)^2 + I - \frac{B^{-1}}{2h} (I - e^{2Bh}) + 2B^{-1} \frac{I - e^{Bh}}{h} \right] \\ &= \sigma_x^2 B^{-2} \left[I - \frac{B^{-1}}{h} \left(\frac{I}{2} + \frac{e^{2Bh}}{2} - e^{Bh} + \frac{I}{2} - \frac{e^{2Bh}}{2} - 2I + 2e^{Bh} \right) \right] \\ &= \sigma_x^2 B^{-2} \left[I + B^{-1} \frac{I - e^{Bh}}{h} \right]. \end{aligned} \tag{S4}$$

In order to explore how the timescales of the driving noise dynamics, i.e., the value of h, impacts the observed covariance matrix Σ, let us first consider the case where h is small. In

that case, we can use a second-order Taylor approximation to rewrite Eq. (S4) as:

$$\Sigma \approx \sigma_x^2\, B^{-2} \left[I - B^{-1} \frac{Bh + (Bh)^2/2}{h} \right] = -h\, \frac{\sigma_x^2}{2}\, B^{-1}. \tag{S5}$$

When h is large, Eq. (S4) suggests:

$$\Sigma \approx \sigma_x^2 B^{-2}. \tag{S6}$$

In other words, it can be seen from Eqs. (S5) and (S6) that when the driving process exhibits fast dynamics, i.e., when h is small, then $\Sigma \propto B^{-1}$, or equivalently $B \propto \Sigma^{-1}$, whereas when the driving process exhibits slow dynamics, we have $\Sigma \propto B^{-2}$, or equivalently $B \propto \Sigma^{-\frac{1}{2}}$. These findings are illustrated using a toy example in Figure S1.

Another way of exploring the timescales of driving and observed dynamics is to reformulate Eq. (2) as:

$$\dot{\mathbf{y}}_t = B^{\alpha} \cdot \mathbf{y}_t + \mathbf{x}_t, \tag{S7}$$

where α is a positive scalar and higher values of α reflect faster observed dynamics $\mathbf{y}_t$. Assuming white Gaussian driving noise $\mathbf{x}_t$, it can be shown that $B \propto \Sigma^{-\frac{1}{\alpha}}$ (e.g., Oku & Aihara, 2018). Hence observing optimal exponents of the SC-FC match closer to 0 in Figure 4 reflects faster timescales of the observed dynamics $\mathbf{y}_t$ as compared to the driving noise $\mathbf{x}_t$.

This interpretation complements and is consistent with Eqs. (S5)-(S6). Indeed, Eq. (S7) suggests that faster processing dynamics of $\mathbf{y}_t$ (i.e., higher exponent of B), given a fixed driving noise $\mathbf{x}_t$ lead to an optimal exponent of Σ that gets closer to 0. On the other hand, Eqs. (S5)-(S6) suggest that slower input dynamics $\mathbf{x}_t$ (i.e., with longer steps h) for fixed processing dynamics $\mathbf{y}_t$ lead to an optimal exponent of Σ closer to -0.5 (coming from -1). So in both cases having an optimal exponent that departs from -1 and goes towards less negative values corresponds to having a decreased ratio between timescales of $\mathbf{y}_t$ and $\mathbf{x}_t$: in one case, the timescale of $\mathbf{y}_t$ decreases ($\mathbf{y}_t$ faster), in the other, the timescale of $\mathbf{x}_t$ increases ($\mathbf{x}_t$ slower).

Optimal exponent for intermediate values of h

In the case of fast input dynamics, we have seen in Eq. (S5) that $B \propto \Sigma^{-1}$, and hence the optimal exponent β is -1. When the input gets slower (i.e., larger values of h), $B \propto \Sigma^{-2}$ and hence the optimal exponent β is -1/2 (Eq. (S6)). To explore the value of the optimal exponent for intermediate values of h, we used a toy example with $M = 10$ variables, $T = 100000$ time points, a minimal step size $dt = 0.005$, and a random (symmetric and stable) structural connectivity matrix B. Figure S1 shows the optimal value of the exponent β for different values of h, averaged over 200 trials.

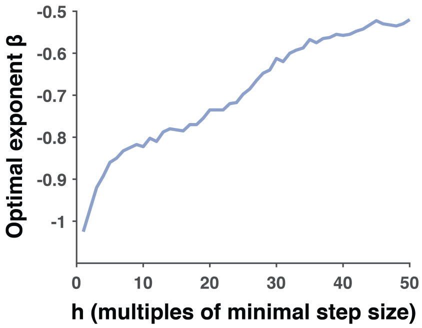

Figure S1. Impact of step size on the value of the optimal exponent.

It can be seen that as expected in the cases of low and high values of h (i.e., $h = 1 * dt$ and $h = 50 * dt$), the optimal exponent is found to be equal to -1 and -1/2, respectively. Moreover, for intermediate values of h, this toy example confirms that the optimal exponent is found to smoothly go from -1 to -1/2 when the value of h increases.

Regularized versions of the precision matrix

Inverting the correlation matrix might be an unstable operation especially when it is computed from few time points (Brier et al., 2015). Therefore, we considered a regularized version of the precision matrix. The regularization used in the results of the main manuscript (Figures 2&3) relies on the Tikhonov regularization. This consists in adding a full-rank regularization term to the correlation matrix before performing the inversion. In other words, the Tikhonov regularized precision matrix is equal to $(\Sigma + \gamma \cdot \mathbb{I})$, where Σ is the empirical correlation matrix, $\mathbb{I}$ is the identity matrix and γ is a parameter that was optimized by minimizing the distance between regularized subject precision matrices and the unregularized group precision matrix. More precisely, we followed the procedure described in Pervaiz et al. (2020) that consists in (i) for a given value of γ compute the N regularized subject precision matrices, (ii) compute the distance between these N matrices and a unique unregularized group precision matrix (after taking the square of all matrix entries), and (iii) choose the value of γ that minimizes the sum of all N distances. The parameter γ was varied between 0 and 1 with 0.02 increments, and its optimal value for different values of functional time series is shown in Figure S2.

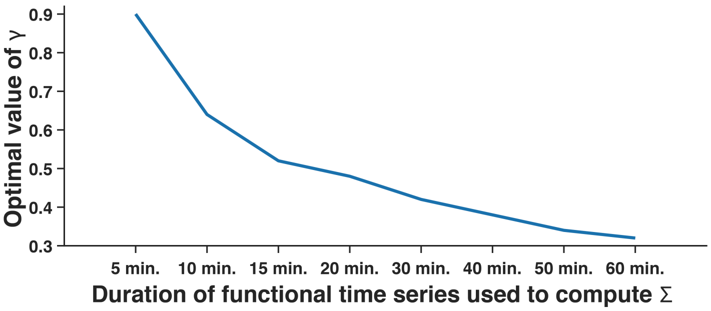

Figure S2. Optimal value of Tikhonov regularizing parameter γ as a function of the functional time series length used to compute the correlation matrix Σ.

As expected, when more time points are used to compute the correlation matrix, the optimal value of γ decreases since less regularization is required. These optimal values of γ were considered when using less than one hour of functional data to compute regularized precision matrices in Figure 2C. When using more than one hour of data, time series from different subjects had to be concatenated and in that case we considered $\gamma = 0.32$ to compute regularized precision matrices in Figure 2C.

SUPPLEMENTARY MATERIAL - RESULTS

Computing SC-FC match using variations of the SC matrix

When exploring the link between SC and FC, the match between these two matrices is often computed only over non-zero SC entries (e.g., Honey et al., 2009). We did not restrict our analyses to non-zero entries mainly because it requires to set a threshold under which an SC entry is considered to be zero and we wanted our results to be as general as possible. To explore the impact of considering such a threshold, we repeated in Figure S3B,C the analysis of Figure 2C but considering only non-zero SC entries to evaluate the SC-FC match. Then, the results shown in the main manuscript were obtained using SC matrices with entries normalized by the volumes of starting and ending ROIs. To further support robustness of our results, we also repeated the analysis of Figure 2C using SC matrices with entries that were not normalized by the corresponding ROIs' volumes (Figure S3D).

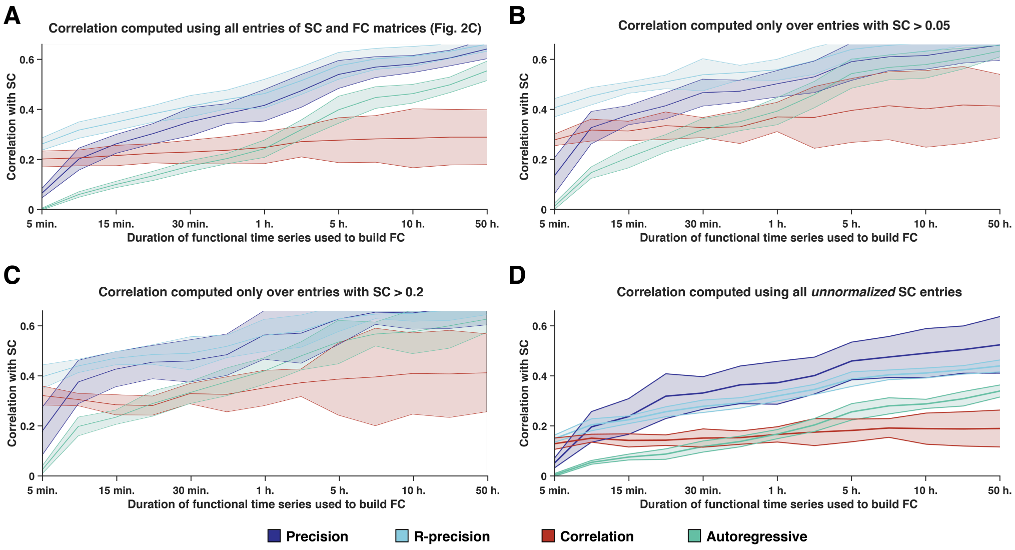

Figure S3. SC-FC match for four FC metrics as a function of data duration to compute FC metrics. (A) The match is computed using all SC and FC entries, similarly to what is presented in Figure 2C, (B) the match is computed using only SC (and corresponding FC) entries that are higher than 0.05, (C) the match is computed using only SC (and corresponding FC) entries that are higher than 0.2, and (D) the match is computed using SC with entries that were not normalized by starting and ending ROIs' volumes.

It can be seen from Figure S3B,C that our main conclusions hold even when computing the SC-FC match only over non-zero SC entries, using two different thresholds. In particular, regularized precision-based FC still outperforms other FC metrics when few data

points are available. Interestingly, there is an overall increase of the correlation-based SC-FC match when only non-zero SC entries are considered: using one hour of data, this match is equal to 0.24 when all entries are used (Figure S3A), it is equal to 0.37 when considering only SC entries higher than 0.05 (Figure S3B), and it is equal to 0.38 when considering only SC entries higher than 0.20 (Figure S3C). Finally, Figure S3D shows that the main results are reproduced when considering unnormalized SC matrices, even if the overall SC-FC match is lower as compared to other cases. This suggests that normalizing SC entries by corresponding ROIs' volumes is a meaningful operation in the context of SC-FC comparisons.

Linking the optimal exponent and behavioral measures

The procedure described in Figure 4 allows to identify the correlation matrix exponent that provides the best match to the SC matrix. As explained in the Supplementary Methods (Eqs. (S1)-(S7)), this metric provides a measure of functional dynamics timescales. We here tested whether this value, computed for each subject, is related to eight HCP behavioral measures including bodily features (age, weight, BMI), arousal measures (sleep quality), cognitive features (fluid intelligence, visual episodic memory, cognitive flexibility), and task performance (relational processing).

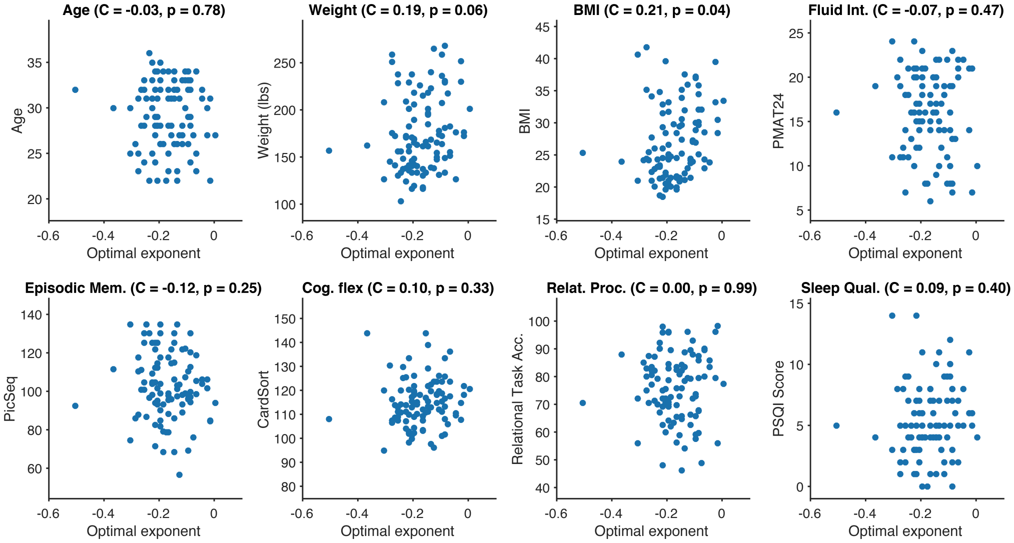

Figure S4. Correlation between the optimal correlation matrix exponent of each subject, and eight HCP behavioral measures: age, weight, BMI, and sleep quality (PSQI score), fluid intelligence (PMAT24), visual episodic memory (PicSeq Unadj), cognitive flexibility (CardSort Unadj), relational processing (relational task accuracy) and sleep quality (PSQI score).

It can be seen from Figure S4 that none of these measures seems to be significantly correlated with the optimal correlation matrix exponent. Note that we have explored the link with other behavioral measures classically used in such applications (e.g., Li et al., 2019; Liégeois et al., 2019), and did not find links that survived correction for multiple comparisons. Future work might explore whether the optimal exponent in specific networks is a better behavioral marker, or if multivariate approaches (e.g., canonical component analy-

sis) might help revealing a link between functional timescales as captured by the optimal exponent and behavior.

Impact of repetition time (TR)

In order to evaluate the impact of the repetition time, we reproduced the analysis of Figure 2C by removing one functional time point every two functional time points (corresponding to an effective TR = 1.44 *sec*), and two functional time points every three functional time points (correponding to an effective TR = 2.16 *sec*).

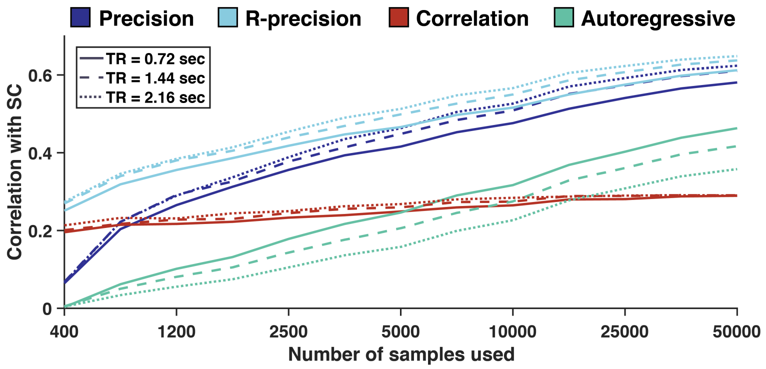

Figure S5. Impact of TR on the (average) match between SC and FC evaluated from the precision matrix (dark blue), the regularized precision matrix (light blue), the correlation matrix (red) and the autoregressive matrix (green) of unfiltered fMRI time-series. We considered TR = 0.72 *sec*, TR = 1.44 *sec*, and TR = 2.16 *sec*.

The results are shown in Figure S5 and suggest that using the same number of fMRI time points but with a larger repetition time (TR), precision-based FC and correlation-based FC estimates have a better match to SC whereas AR-based FC is less correlated with SC. This is explained by the fact that fMRI time points carry less redundant information when the sampling period TR increases, which is beneficial for the estimation of the correlation and precision matrices, but penalizes the estimation of the AR-based metric because it exploits autocorrelation of time series. Obviously, considering longer repetition times also comes at the price of longer scanning sessions for a given number of time points.

Subject-level optimal exponent using short acquisition times

The results of Figure 4 were computed using data concatenated over all subjects. In practice, such amount of data is not always available and we here provide the corresponding curves when using only the first HCP run (i.e., 1200 time points) for the three first HCP subjects. Since matrix inversion operations might be unstable when using such small amount of data, we used the regularization procedure described in Figure S2 with $\gamma = 0.52$ which is the optimal regularization parameter when using 1200 time points.

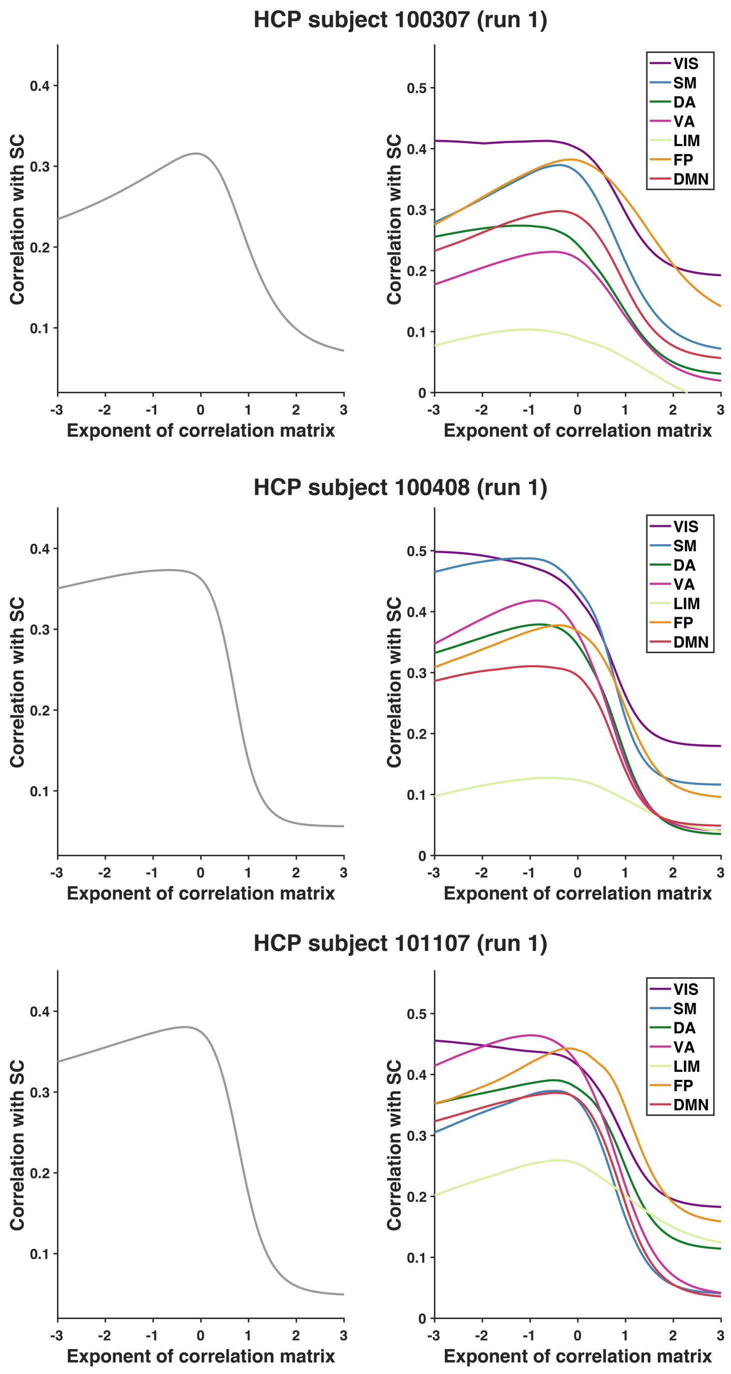

Figure S6. Correlation between SC and FC evaluated from various exponents of the correlation matrix computed from the first functional run in three HCP subjects, i.e., using 1200 time points. (Left) Using whole-brain data. (Right) Using only data in the 7 resting-state networks of B. T. T. Yeo et al. (2011).

It can be seen that when using whole-brain data (left panels in Figure S6), subject-level curves are similar to the group-level curve shown in Figure 4A even if the optimal ex-

ponents seem to have smaller absolute values. Within resting-state networks, the overall shape also seems to be similar at the subject-level (right panels in Figure S6) as compared to the group level (Figure 4A), with common features such as a high SC-FC match in the visual network and a low SC-FC match in the limbic network. Future work might explore distribution of these curves across subjects and test whether they encode behavioral information, as presented in Figure S4 at the whole-brain level.

Average functional and structural matrices

Figure S7 shows different average structural and functional connectomes (upper row), together with the corresponding distributions of entries (lower row).

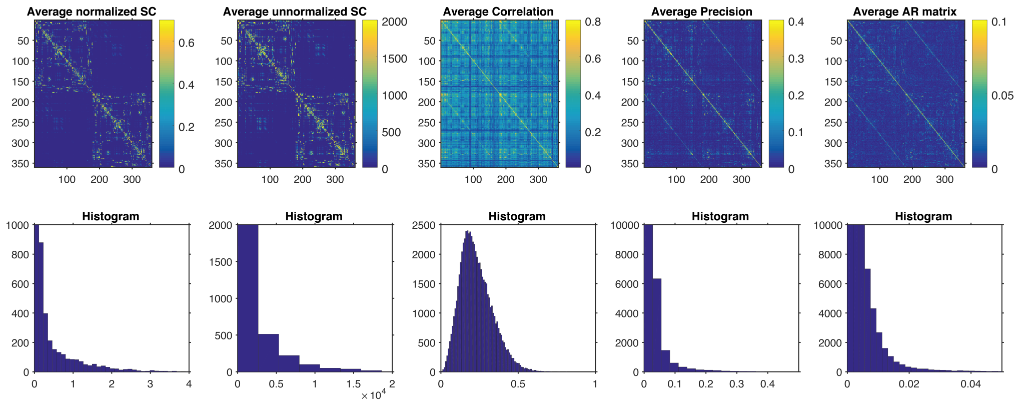

Figure S7. (Upper row) From left to right: average structural connectome (number of streamlines normalized by starting and ending ROIs' volumes), average unnormalized structural connectome, average correlation matrix, average precision matrix, and average autoregressive (AR) matrix. (Lower row) Corresponding histogram of entries.

Impact of averaging effects

One question we might have when observing results of Figure 2C is whether averaging functional and structural connectomes over different subjects might boost the SC-FC match. To test this, we computed this match using four runs of functional data ($\sim$ 1 hour) in three cases: (i) the four runs come from the same subject and are compared to the subject's SC matrix (as in Figure 2B), (ii) the four runs come from two different subjects chosen at random (first two runs are the first two runs of one subject, and last two runs are the last two runs of the other subject) and are compared to the corresponding averaged SC matrix, and (iii) the four runs come from four different subjects chosen at random and are compared to the corresponding averaged SC matrix. Experiment (i) was performed for all 100 subjects, and experiments (ii) and (iii) were repeated 100 times.

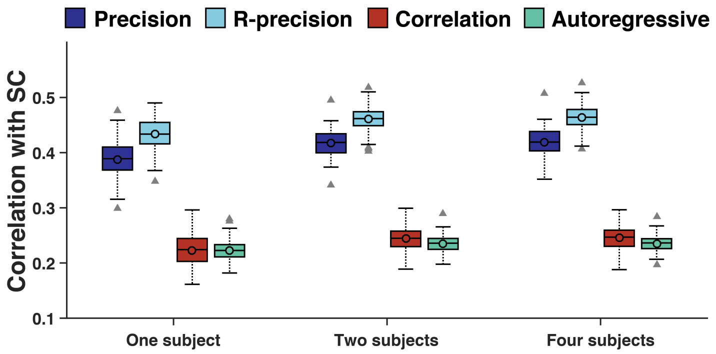

Figure S8. SC-FC match using four runs of functional data in three cases: (i) the four runs come from the same subject, (ii) the four runs come from two different subjects chosen at random, and (iii) the four runs come from four different subjects chosen at random.

Results shown in Figure S8 indeed suggest that averaging functional and structural connectomes slightly increases the SC-FC match as compared to the case where data coming from one subject is used, which might partly explain the increase observed in Figure 2C when using data from several subjects. We finally note that our main findings, i.e. the advantage of (regularized) precision-based FC over other FC metrics in the context of SC-FC comparisons, is reproduced in the three configurations presented in Figure S8.
